# Clinical Characteristics and Management of Drug-Induced Lupus Caused by Tumor Necrosis Factor Inhibitors: A Comprehensive Review

**DOI:** 10.7759/cureus.72522

**Published:** 2024-10-28

**Authors:** Seyedehtina Safaei, Ali Kimiaei, Malik Kasapoglu, Fulya Coşan

**Affiliations:** 1 Internal Medicine, Bahçeşehir University, Istanbul, TUR

**Keywords:** case series, dil, drug-induced lupus, sle, tnf inhibitors

## Abstract

Tumor necrosis factor (TNF) inhibitor (TNFi) therapy, commonly used to treat autoimmune conditions, such as ankylosing spondylitis (AS), rheumatoid arthritis, and psoriatic arthritis, has been associated with the development of drug-induced lupus (DIL). While the incidence of TNFi-induced DIL is relatively rare, it typically presents with mild symptoms and can often be managed with continued therapy or medication switches. This review explores the clinical characteristics, management strategies, and outcomes of TNFi-induced DIL through a comprehensive examination of cases reported in the literature. The findings suggest that most patients develop anti-double-stranded DNA (anti-dsDNA) positivity without severe clinical manifestations, and major organ involvement is uncommon. Treatment strategies vary; some patients can maintain TNFi therapy without disease exacerbation, while others require alternative treatments. Further research is needed to refine management protocols and improve patient outcomes.

## Introduction and background

Drug-induced lupus (DIL) is an autoimmune disorder triggered by certain medications, resulting in clinical features that resemble those of systemic lupus erythematosus (SLE) in various respects [1]. It is usually linked to mild symptoms, mainly mucocutaneous and articular, but some cases show major organ involvement [1]. Although there are no universally accepted diagnostic criteria for DIL, diagnosis typically involves the following parameters: the administration of one or more drugs known to be linked to this condition for a minimum of one month, the emergence of at least one clinical sign characteristic of SLE, and the resolution of symptoms following the discontinuation of the medication [1-3].

While a variety of drugs have been associated with DIL, this review will concentrate specifically on tumor necrosis factor (TNF) inhibitors (TNFi), which are commonly utilized in rheumatology and prescribed for patients experiencing high disease activity. Information regarding the management and follow-up of DIL related to TNFi is scarce and mainly based on individual case reports, indicating a lack of comprehensive studies despite the existence of reported cases. In the majority of instances, the recommended approach is to discontinue TNFi. However, in certain scenarios where alternative treatments are limited or unavailable, it may be appropriate to continue or reintroduce TNFi.

This review seeks to compile the existing literature on TNFi-induced DIL, emphasizing clinical features, management approaches, and outcomes from reported cases to enhance understanding of this condition and its relevance in clinical practice.

## Review

Methods

A comprehensive literature review was conducted to investigate cases of DIL associated with TNF inhibitor (TNFi) therapy. The search was performed using the PubMed database, covering publications from the database's inception to March 2024. The search strategy incorporated the following keywords: "drug-induced lupus", "DIL", and "TNF inhibitors (TNFi)".

To refine the search, we applied specific filters, focusing exclusively on case reports and case series, as these study types provide detailed clinical insights into individual patients or small groups of patients. Review articles, randomized controlled trials, and studies that did not directly report cases of DIL associated with TNFi were excluded.

The information extracted from the selected studies included patient demographics, underlying conditions, the specific TNFi used, clinical manifestations, serological findings (such as anti-double-stranded DNA (anti-dsDNA positivity), treatment modifications (whether TNFi therapy was discontinued or continued), and patient outcomes.

By analyzing these cases, our review aimed to summarize common patterns in clinical presentation, explore various management strategies, and assess outcomes to inform future clinical decisions and research efforts.

Ethical considerations and acknowledgments

As this study is a review of existing literature, it does not directly involve human subjects and therefore does not necessitate ethical approval. The authors declare no financial incentives or conflicts of interest.

Results

The search identified 54 case reports and case series, documenting a total of 75 patients who developed DIL during or following TNFi therapy. A summary of these cases is presented in Table 1.

**Table 1:** Summary of case reports and case series in the literature. RA: rheumatoid arthritis; CD: Crohn's disease; UC: ulcerative colitis; HS: hidradenitis suppurativa; PsO: psoriasis; AEGCG: annular elastolytic giant cell granuloma; AS: ankylosing spondylitis; GPA: granulomatosis with polyangiitis; SS: Sjögren's syndrome; MCTD: mixed connective tissue disease; AOSD: adult-onset Still's disease; IgG: immunoglobulin G; IgM: immunoglobulin M; RF: rheumatoid factor; ACPA: anti-citrullinated protein antibodies; PAIgG: platelet-associated immunoglobulin G; ANCA: anti-neutrophil cytoplasmic antibodies; ANA: antinuclear antibodies; HACA: human anti-chimeric antibodies; NA: not available; anti-dsDNA: anti-double stranded DNA antibodies; ENA: extractable nuclear antigens; anti-SSB: anti-Sjögren's syndrome B antibodies; anti-RNP: anti-ribonucleoprotein antibodies; anti-SS-B/La: anti-Sjögren's syndrome B/La antibodies; U1RNP: U1 ribonucleoprotein; anti-SSA: anti-Sjögren's syndrome A antibodies; anti-Ro: anti-Ro antibodies; SLE: systemic lupus erythematosus; -: negative; +: positive

Study	Patient count	Pre-existing disease	Symptoms	Laboratory	ANA	Anti-dsDNA	ENA	Anti-histone	Major organ involvement	Responsible drug	Drug changes	Prognosis
Waitayangkoon et al. 2023 [4]	1	RA	Fever, sore throat, productive cough, bilateral lower extremity pain	Anti-cardiolipin IgG and IgM, decreased C3 and C4	+	+	Anti-SSA/Ro	+	Nephritis, cardiomyopathy	Adalimumab	Discontinuation	Chronic SLE
Thiriveedi et al. 2021 [5]	1	CD	Pleuritic chest pain, dyspnea, arthralgia	RF	+	+	-	+	Pericarditis	Infliximab	Discontinuation	Improved with Discontinuation
Pan et al. 2021 [6]	1	RA	Hair loss, body edema, cheek erythema, involuntary handshaking	Decreased C3, RF	+	+	-	+	Nephritis	Anbainuo (biosimilar to etanercept)	Discontinuation	Chronic SLE
AlBloushi et al. 2020 [7]	1	UC	Uveitis	NA	NA	NA	NA	NA	NA	Adalimumab	Discontinuation	Improved with Discontinuation
Yoshikawa et al. 2020 [8]	1	RA	Urticaria, rash	RF, ACPA, PAIgG, decreased C3 and C4	-	+	Anti-SS-A	-	Skin (bullous pemphigoid)	Adalimumab	Discontinuation	Improved with Discontinuation
Loebenstein et al. 2020 [9]	1	CD	Fatigue, myalgias, arthralgia		+	+	-	-	No	Infliximab	Change to another TNFi	Improved with a Change to Another TNFi
Turk et al. 2020 [10]	1	HS	Arthralgia		+	-	-	+	No	Adalimumab	Discontinuation	Improved with Discontinuation
Stein et al. 2018 [11]	1	PsO	Arthralgia		+	+	NA	NA	No	Adalimumab	Discontinuation	Improved with Discontinuation
Lomicova et al. 2017 [12]	1	PsO	Myalgia with paresthesia		+	-	NA	-	No	Adalimumab	Discontinuation	Improved with Discontinuation
Saka et al. 2017 [13]	1	RA	Fatigue, appetite loss, severe renal dysfunction, massive proteinuria		+	-	-	-	Nephritis	Golimumab	Discontinuation	Chronic SLE
Mudduluru et al. 2017 [14]	1	CD	Arthralgia		+	+	-	+	No	Certolizumab	Change to another TNFi	Improved with Discontinuation
Haimovic et al. 2017 [15]	1	AEGCG	Arthralgia		+	+	-	-	No	Adalimumab	Discontinuation	Improved with Discontinuation
Akgul et al. 2014 [16]	1	AS	Fatigue, arthritis, fever.		+	+	-	-	No	Infliximab	Discontinuation	Improved with Discontinuation
Harnett et al. 2014 [17]	1	UC	Pleuritic chest pain, dyspnea		+	+	-	-	Cardiac tamponade	Infliximab	Discontinuation	Improved with Discontinuation
Dang et al. 2014 [18]	1	PsO	Photosensitivity, arthritis, mouth ulcers		+	-	-	-	No	Infliximab	Discontinuation	Improved with Discontinuation
Santiago et al. 2013 [19]	1	AS	Malaise, dyspnea, chest pain		+	+	-	+	No	Infliximab	Change to another TNFi (Etanercept)	Improved with a Change to Another TNFi
Yahya et al. 2013 [20]	1	PsO	Palpable purpura, hypertension	Decreased C3, RF, type II mixed cryoglobulins	+	+	Anti-SSB	-	Nephritis	Etanercept	Discontinuation	Improved with Discontinuation
Farkas et al. 2013 [21]	1	CD	Arthritis		+	+	Anti-RNP, anti-Smith, anti-SSA/Ro, anti-SS-B/La	+	No	Infliximab	Discontinuation	Improved with Discontinuation
Adler et al. 2013 [22]	1	GPA	Fever, polyarthritis	Leukopenia, ANCA, decreased C3 and C4	+	+	-	-	Pericarditis, endocarditis	Infliximab	Discontinuation	Improved with Discontinuation
Guerin et al. 2012 [23]	1	RA	Myalgia, asthenia, synovitis, arthritis, rheumatoid nodules, purplish infiltrated erythematous plaques		+	+	-	-	No	Adalimumab	Discontinuation	Improved with Discontinuation
Liggi et al. 2011 [24]	1	CD	Myalgia, arthritis, morning stiffness		+	+	-	-	No	Infliximab	Discontinuation	Improved with Discontinuation
Vannucchi et al. 2011 [25]	1	CD	Right superior quadrantopsy, headache	Anti-cardiolipin IgM, Anti-phospholipid IgM	+	+	-	-	CNS vasculitis	Adalimumab	Discontinuation	Improved with Discontinuation
Ye et al. 2011 [26]	2	RA	1: Arthritis; 2: Oral ulcers, arthritis, synovitis		-	+	-	-	No	1: Adalimumab; 2: Infliximab	Change to another TNFi (Etanercept)	Improved with a Change to Another TNFi
Chogle et al. 2011 [27]	1	RA	Erythematous plaques		+	-	-	-	No	Etanercept	Discontinuation	Improved with Discontinuation
Williams et al. 2011 [28]	1	RA	Pruritic photo-distributed rash		+	-	Anti-SSA/Ro, anti-SS-B/La	-	No	Etanercept	Change to another TNFi (Golimumab)	Improved with a Change to Another TNFi
Caramaschi et al. 2010 [29]	1	PsO	Fever, polyarthritis		+	+	-	-	No	Infliximab	Discontinuation	Improved with Discontinuation
Luong et al. 2010 [30]	1	AS	Arthralgia, papular eruption	Decreased C3 and C4	+	+	-	-	No	Infliximab	Change to another TNFi (Etanercept)	Improved with a Change to Another TNFi
Zella et al. 2009 [31]	1	CD	Arthralgia	HACA	+	+	-	-	No	Infliximab	Discontinuation	Improved with Discontinuation
Neradova et al. 2009 [32]	1	RA	Photosensitivity, butterfly rash, fatigue	Decreased C3 and C4	+	-	-	-	Pericarditis, nephritis	Etanercept	Discontinuation	Improved with Discontinuation
Kocharla et al. 2009 [33]	1	CD	Arthralgia, facial rash, photosensitivity, oral ulcers, fatigue		+	+	-	NA	No	Infliximab	Discontinuation	Improved with Discontinuation
Bodur et al. 2009 [34]	1	AS	Arthralgia, visual impairment, cough, weakness, anorexia, fatigue		+	-	Anti-Smith	-	No	Infliximab	Discontinuation	Improved with Discontinuation
Mounach et al. 2008 [35]	1	AS	Arthralgia		+	+	+	+	No	Infliximab	Discontinuation	Improved with Discontinuation
Abunasser et al. 2008 [36]	1	AS	Pleuritic chest pain, dyspnea, pleural effusion		+	-	-	+	No	Etanercept	Discontinuation	Improved with Discontinuation
Havel et al. 2008 [37]	1	RA	NA	NA	NA	NA	NA	NA	NA	Adalimumab	Discontinuation	Improved with Discontinuation
Martin et al. 2008 [38]	1	RA	Rash		+	-	-	+	No	Adalimumab	Discontinuation	Improved with Discontinuation
Page et al. 2008 [39]	1	UC	Pleural effusion, fever, myalgia		+	-	-	+	No	Infliximab	Discontinuation	Improved with Discontinuation
Manosa et al. 2008 [40]	2	CD	NA	NA	NA	NA	NA	NA	NA	Adalimumab	NA	NA
Kang et al. 2006 [41]	1	RA	Fatigue, malaise, fever, myalgia, oral ulcers, gingivitis, anorexia	RF	+	+	Anti-SSA/Ro	-	Pneumonitis	Etanercept	Discontinuation	Chronic SLE
van Rijthoven et al. 2006 [42]	1	RA	Arthritis, fever, photosensitivity, rash, oral ulcers	RF	+	+	-	-	No	Adalimumab	Discontinuation	Chronic SLE
Chadha et al. 2006 [43]	1	RA/SS	Fever, malar rash, synovitis	RF, decreased C3 and C4	+	+	Anti-SSA/Ro, anti-SSB/La	+	Endocarditis	Infliximab	Discontinuation	Improved with Discontinuation
Perez-Garcia et al. 2006 [44]	1	AS	Malaise, fever, myalgias, arthritis		+	+	-	-	No	Infliximab	Discontinuation	Improved with Discontinuation
Pallotta et al. 2006 [45]	1	PsO	Fever, pleural effusion, arthralgia		+	-	-	+	No	Infliximab	Discontinuation	Improved with Discontinuation
Benucci et al. 2005 [46]	1	RA	Synovitis, myalgia, rash, pericardial and pleural effusion		+	+	-	-	No	Infliximab	Discontinuation	Improved with Discontinuation
Christopher-Stine et al. 2003 [47]	2	MCTD	1: Fever, malaise, anemia, arthritis; 2: Flank pain, nausea, vomiting, arthralgias, myalgias	2: Decreased C3 and C4	+	+	1: Anti-SS-A/Ro, U1RNP; 2: U1RNP	-	No	1: Etanercept; 2: Infliximab	Discontinuation	Improved with Discontinuation
Sarzi-Puttini et al. 2003 [48]	1	CD	Arthralgia, malar rash	ANCA	+	+	-	-	No	Infliximab	Discontinuation	Chronic SLE
Debandt et al. 2003 [49]	3	RA	1: Myalgia, fatigue, rash; 2: Rash; 3: Chest pain, pericarditis, arthralgia, photosensitivity, malar rash	1: RF, decreased C4, anticardiolipin antibodies; 2: RF; 3: RF, Anticardiolipin	+	1, 3: +	-	-	3: Pericarditis	1, 2: Etanercept; 3: Infliximab	1,2: Discontinuation; 3: Change to another TNFi (Etanercept)	1, 2: Improved with Discontinuation; 3: Improved with a Change to Another TNFi
Ali et al. 2002 [50]	1	CD	Arthritis		+	+	-	+	No	Infliximab	Discontinuation	Improved with Discontinuation
Favalli et al. 2002 [51]	1	RA	Fever, arthralgia, myalgia, malaise, rash		+	+	-	NA	No	Infliximab	Discontinuation	Improved with Discontinuation
Shovman et al. 2018 [52]	3	1, 3: UC; 2: CD	1: Thrombocytopenia; 2: Arthritis; 3: Pleural effusion, pericardial effusion, ascites	2: Anticardiolipin	+	+	-	2: +	No	Infliximab	Discontinuation	Improved with Discontinuation
Diaz et al. 2012 [53]	3	RA	1: Fever, malaise, arthralgias, alopecia, urticarial vasculitis; 2: Arthralgias; 3: Vasculitis, arthralgia, anorexia, fever	3: Decreased C3 and C4	+	+	Anti-SS-A/Ro	-	No	1, 2: Etanercept; 3: Adalimumab	Discontinuation	Improved with Discontinuation
Soforo et al. 2010 [54]	6	1, 2, 3, 5, 6: RA; 4: PsO	1: Malar rash, photosensitivity, arthritis, pericarditis; 2: Photosensitivity, arthritis, optic neuritis; 3: Malar rash, discoid rash, photosensitivity, arthritis; 4: Malar rash, photosensitivity, arthritis; 5: Malar rash, photosensitivity, pericarditis; 6: Arthritis, photosensitivity	1: Anticardiolipin	+	2, 4, 6: +	-	-	No	1, 2, 4, 5, 6: Etanercept; 3: Infliximab	Discontinuation	Improved with Discontinuation
Diri et al. 2007 [55]	3	RA	1: Dyspnea, cough, fatigue, pleural effusion; 2: Dyspnea, fever, pleural effusions; 3: Chest pain, fever, pleural effusion	3: Decreased C3 and C4	+	2, 3: +	-	-	No	Infliximab	Discontinuation	Improved with Discontinuation
Costa et al. 2008 [56]	3	1: RA; 2: AOSD; 3: CD	1: Arthralgia, fatigue, fever, malar rash; 2: Arthralgia, fever, rash; 3: Fatigue, rash, arthralgia, malaise	1: Decreased C4	+	1, 3: +	-	+	No	1: Adalimumab; 2: Etanercept; 3: Infliximab	Discontinuation	Improved with Discontinuation
Shakoor et al. 2002 [57]	4	RA	1: Malaise, fevers, polysynovitis; 2: Discoid rash; 3: Facial erythema, hypertension, peripheral edema, arthralgia; 4: Pleuritic chest pain, malar rash, skin erythema	4: Decreased C3 and C4	+	+	4: Anti-Smith, anti-RNP	+	No	Etanercept	Discontinuation	Improved with Discontinuation

Pre-existing Disease

The distribution of rheumatological conditions among the 75 patients was as follows: rheumatoid arthritis (RA) accounted for 46.66% (35 patients), Crohn's disease (CD) for 18.66% (14 patients), psoriasis (PsO) for 9.33% (seven patients), and ankylosing spondylitis (AS) for 9.33% (seven patients). Ulcerative colitis accounted for 6.66% (five patients), mixed connective tissue disease for 2.66% (two patients), and hidradenitis suppurativa, annular elastolytic giant cell granuloma, granulomatosis with polyangiitis, and adult-onset still's disease (AOSD) each for 1.33% (one patient each). Additionally, both RA and Sjögren's syndrome were reported in 1.33% (one patient).

Clinical Manifestations and Major Organ Involvement

A wide array of clinical symptoms was recorded among the patients, highlighting the heterogeneous nature of DIL. Common symptoms included fever, arthralgia, and myalgias, with several patients also presenting with pleuritic chest pain and respiratory difficulties. Notably, dermatological manifestations such as rash and photosensitivity were prevalent, reflecting the autoimmune nature of DIL. Major organ involvement was observed in 13 patients (17.33%). Among these patients, five presented with pericarditis, four with nephritis, one with endocarditis, one with pneumonitis, and one with bullous pemphigoid of the skin. Therefore, the most commonly involved major organ appears to be the heart (pericarditis), followed by the kidneys (nephritis).

Laboratory Findings

The laboratory findings corroborated the clinical presentations, as antinuclear antibodies (ANA) were positive in most cases. Additionally, anti-double-stranded DNA (anti-dsDNA) antibodies were frequently detected. Other relevant tests showed varying positivity for extractable nuclear antigens (ENA), including anti-SSA/Ro and anti-SSB/La, as well as anti-histone antibodies and complement levels (C3 and C4).

Responsible Agents

Among the 75 patients, the following agents were identified as responsible for the development of DIL: infliximab in 45.3% (34 cases), adalimumab in 22.7% (17 cases), etanercept in 28.0% (21 cases), and certolizumab in 1.3% (one case). Additionally, anbainuo and golimumab were each responsible for 1.3% (one case each).

Management and Prognosis

In managing DIL in the 75 identified cases, the following strategies were employed: discontinuation of TNF inhibitors (TNFi) in 88.0% (66 cases), switching to another TNFi in 9.3% (seven cases), and management strategy data were unavailable in 2.7% (two cases). Regarding the prognosis of these patients, chronic systemic lupus erythematosus (SLE) was observed in 8.0% (six cases). Significant improvement was noted following treatment discontinuation in 81.3% (61 cases), while improvement was observed after switching to another TNFi in 8.0% (six cases). Prognostic data were unavailable in 2.7% (two cases).

Discussion

The association between DIL and TNFi therapy presents a complex clinical challenge, as evidenced by the diverse cases reported in the literature. This review aimed to synthesize findings from multiple case reports to enhance our understanding of the clinical characteristics, treatment strategies, and outcomes associated with DIL in patients receiving TNFi therapy.

Typically, DIL is associated with mild clinical symptoms, as indicated by several studies that have demonstrated only mucocutaneous and articular manifestations [1]. However, in some cases, there is evidence of major organ involvement [4-6,8,13,17,20,22,25,32,41,43,49]. Major organ involvement was observed in 13 patients (17.33%). Among these patients, five presented with pericarditis, four with nephritis, one with endocarditis, one with pneumonitis, and one with bullous pemphigoid. Thus, the most commonly affected major organ appears to be the heart (pericarditis), followed by the kidneys (nephritis).

The high frequency of autoantibody induction, including ANA and anti-dsDNA antibodies, in patients treated with TNFi agents is well documented [58,59]. The administration of TNF-α antagonists can lead to elevated titers of ANA in patients who already have positive ANA serology, while new-onset positive ANA may also develop in previously negative ANA patients receiving TNF-α inhibitors [58]. Other antibodies, such as anti-histone antibodies, anticardiolipin antibodies, and anti-Smith antibodies, have also been reported [58,60,61]. Additionally, positive ENA may develop in patients on anti-TNF-α agents [58].

Studies have estimated the incidence of DIL erythematosus to be 0.19%-0.22% for infliximab, 0.18% for etanercept, and 0.10% for adalimumab during treatment [60,62-64]. In our review of 75 patients, adalimumab was identified as the most common agent associated with the development of DIL, accounting for 22.7% of cases. Although these biological agents share similar mechanisms of action, understanding the differences in the risk of DIL associated with each drug is challenging. These differences may be attributed to variations in molecular structure, bioavailability, binding affinity, immunogenicity, and metabolism [19,44].

In managing DIL among the identified cases, the predominant strategy involved discontinuing the TNFi. This approach reflects a common practice in the clinical management of DIL. A smaller subset of patients opted to switch to another TNFi, suggesting that some clinicians may consider continuity of therapy when feasible, particularly in patients with limited treatment options, such as those with AS [9,14,19,26,28,30,49].

The prognosis for patients diagnosed with DIL appears generally favorable following the discontinuation of TNFi. Significant improvements in symptoms were noted in many cases after stopping treatment, indicating that DIL is often reversible. However, instances of chronic SLE development were observed, emphasizing the potential for long-term complications in some patients [4,6,13,41,42,48]. These findings underscore the importance of vigilant monitoring and individualized management strategies for patients experiencing DIL, as careful assessment can lead to better outcomes.

Limitations

This study has several limitations. The reliance on case reports may introduce bias, as these reports often focus on unique cases that may not represent the broader patient population. Additionally, the absence of standardized diagnostic criteria for DIL complicates the generalizability of the findings. Furthermore, variability in treatment approaches and follow-up among studies limits the ability to draw definitive conclusions regarding optimal management strategies. Future research utilizing more robust methodologies is essential to provide clearer guidelines for managing DIL associated with TNFi therapy.

## Conclusions

In conclusion, this review emphasizes the challenges associated with DIL in patients receiving TNFi therapy. In particular, treatment modification options for conditions such as AS are often limited, which may necessitate the continuation of ongoing therapies despite the associated risk of DIL. The findings highlight the need for careful monitoring and tailored management strategies to balance the benefits of TNFi therapy against the potential risks of developing DIL. To enhance patient outcomes, further research is essential, including larger-scale studies, comprehensive follow-up, and the establishment of standardized treatment protocols. These initiatives will be crucial for improving our understanding of DIL and optimizing management strategies for affected patients.
